# EVALUATION OF THE EXTRACELLULAR MATRIX OF INJURED SUPRASPINATUS IN RATS

**DOI:** 10.1590/1413-785220162401146706

**Published:** 2016

**Authors:** Luiz Henrique Oliveira Almeida, Roberto Ikemoto, Ana Maria Mader, Maria Aparecida Silva Pinhal, Bruna Munhoz, Joel Murachovsky

**Affiliations:** 1 Faculdade de Medicina do ABC, Santo André, SP, Brazil.

**Keywords:** Collagen, Rats, Wistar, Extracellular Matrix

## Abstract

**Objective:**

To evaluate the evolution of injuries of the supraspinatus muscle by immunohistochemistry (IHC) and anatomopathological analysis in animal model (Wistar rats).

**Methods:**

Twenty-five Wistar rats were submitted to complete injury of the supraspinatus tendon, then subsequently sacrificed in groups of five animals at the following periods: immediately after the injury, 24h after the injury, 48h after, 30 days after and three months after the injury. All groups underwent histological and IHC analysis.

**Results:**

Regarding vascular proliferation and inflammatory infiltrate, we found a statistically significant difference between groups 1(control group) and 2 (24h after injury). IHC analysis showed that expression of vascular endothelial growth factor (VEGF) showed a statistically significant difference between groups 1 and 2, and collagen type 1 (Col-1) evaluation presented a statistically significant difference between groups 1 and 4.

**Conclusion:**

We observed changes in the extracellular matrix components compatible with remodeling and healing. Remodeling is more intense 24h after injury. However, VEGF and Col-1 are substantially increased at 24h and 30 days after the injury, respectively. ***Level of Evidence I, Experimental Study.***

## INTRODUCTION

The injuries of the rotator cuff muscles are among the injuries that most commonly cause pain and functional impotence in adults' shoulders. Its prevalence is high and ranges from 7 to 40%, increasing with age.^1^

The cause of these injuries is still a matter of controversy,^2^ and vascularization, degeneration and trauma patterns are the main responsible factors identified for triggering failure of the muscles fibers.^1^

Recent studies evaluating the supraspinatus pathological samples collected during surgery and submitted to immunohistochemical studies demonstrated vascular proliferation in the distal portion of the injury, contradicting the hypovascularization hypothesis.^3^ The vascularization found did not correlated with age, gender or time of pain, and increased vascularization was found on the edges of injuries.^3^ It is believed that this hypervascularization arises from an inflammatory response of the foreign body in response to degenerative and progressive changes of the tendon.

Clinical studies using magnetic resonance imaging (MRI) showed that fibrosis, muscular atrophy and fatty infiltration are the major consequences of the rotator cuff injury, and these are progressive.^4^

Cytological changes due to intraepithelial rupture of the tendon that forms the rotator cuff have not been fully elucidated so far. Some studies using cadavers or muscle biopsy samples showed a reduction of the diameter of muscle fibers, interfascicular fibrosis, and fatty infiltration.^5^

It is known that changes occur to extracellular matrix components such as collagen and metalloproteases during the process of collapse and regeneration of the rotator cuff. Moreover, some articles describe changes of type I collagen (Col-1), the main collagen constituent of the extracellular matrix of tendons, that due to its dense and parallel fiber arrangement, is the main responsible for the resistance and elasticity of tendons. However, little is known about the molecular alterations that occur during the regeneration process of the rotator cuff.^5^

The choice for the rat model was due to the fact that their scapular girdle is very similar to humans', with coracoacromial arch and rotator cuff muscles, besides ease of handling and cost relative to other models animals such as sheep and primates.^6^

The objective of this study was to evaluate the evolution of the supraspinatus muscle injury by immunohistochemical and pathological analyzes using Wistar rats as animal model.

## MATERIALS AND METHODS

The study was conducted using 25 adult Wistar rats between 17 and 20 weeks of age, which underwent surgery that caused complete injury of the supraspinatus muscle. The rats were provided by the Animal Facility of Hospital Albert Einstein, São Paulo, SP, Brazil. This study was approved by the Ethics Committee on Animal Experimentation of *Faculdade de Medicina do ABC*, São Paulo, SP, Brazil (Ref. CEA Einstein nº 191-06). The entire procedure on the animals took place under general anesthesia. Anesthetic drugs used were ketamine (1.2 ml x weight) and xylazine (0.5 mL x weight), applied intramuscularly in the same insulin syringe. When necessary, the procedure was repeated 40 min later with half the dose previously applied. In the preoperative period, all animals received prophylactic antibiotics for 48h.

All rats were submitted to a superior surgical access way on the right scapular-humeral joint after shaving the area. The supraspinatus muscle containing the complete muscle injury was removed near the greater tubercle of the humerus in all rats. After inducing the injury at the supraspinatus muscle, surgical incision was delimited by planes with 4-0 and 3-0 nylon thread. Postoperatively the animals received analgesia with intramuscular codeine. During the first three days after surgery they received weighted doses of paracetamol diluted in drinking water.

The first five mice were sacrificed immediately after the injury by intravenous potassium chloride. Then, resection of the entire muscle was carried out up to half of their muscle mass. The groups were classified according to the time of sacrifice after rotator cuff injury, each group with five animals. Thus, those sacrificed immediately after the injury were classified as group I. The samples from animals of groups II, III, IV and V were respectively obtained after 24h, 48h, one month and three months after injury.

The tendons were submitted to pathological examination after hematoxylin-eosin staining, and were divided into three areas. The first area covered the edge of the injury to 2 mm medial portion; the second area comprised 2-4 mm medial portion; and the third area covered 4mm medial portion to the supraspinatus muscle myotendinous transition.

### Immunohistochemistry (IHC) analysis 

Three millimeter thick cuts embedded in paraffin and formalin-fixed were deparaffinized and rehydrated. Antigen retrieval was performed by heating the slides at 100°C for 30 min in a 10 mmol/L citrate buffer pH 6.0. The endogenous peroxidase activity was blocked with 3% aqueous hydrogen peroxide for 35 min. 

Regarding primary antibodies, anti-aggrecan (4F4), anti-VEGF (sc-507), anti-heparanase 1 (anti-HPA) (C-20 and IL-6, sc-130 326) (Santa Cruz Biotechnology, CA, USA) and Syn-Anti-CD138 BB4 MCA681 1 (AbD Serotec^(r)^, Bio-Rad Company Co. Oxford, UK), anti-Cat-B and anti-Col1 (C-2456) (Sigma-Aldrich, St. Louis, MO, USA) were used.

Finally, the slides were incubated with streptavidin complex labeled with peroxidase (LSAB^(r)^, DakoCytomation, Glostrup, Denmark) for 30 min. Slides were revealed using 3,3'-diaminobenzidine as a chromogen for 1 min, and then counter stained with hematoxylin. Some samples were incubated with sodium phosphate buffer 1X concentrated from a 50 mmol/L solution instead of the primary antibody as negative control reaction. The presence of brown color was considered positive evidence of cellular protein expression. Histological sections for each specific antibody, according to the manufacturer's protocol, were used as positive control reaction.

### Histomorphometric analysis

The tissues were analyzed using the twelve fields digital quantification (400X), as described in Matos et al.^7^ The mean and standard deviation values ​​were expressed as ItE (expression strength) of triplicate measurements made in all samples carried out by two observers.

### Statistical analysis

Statistical analysis was performed using the Prism5^(r)^ software (GraphPad Software, La Jolla, CA, USA). All variables were considered nonparametric through the Kolmogorov-Smirnov test. The Kruskal-Wallis tests together with the Dunn and Mann-Whitney test were compared and described in means and standard deviations (mean±SD). In all analyzes we adopted the significance level *p*<0.05.

## RESULTS

**Figure 1:**
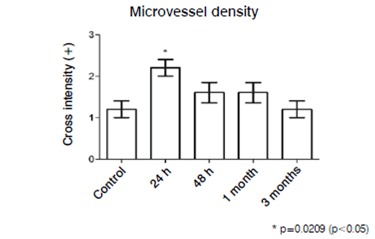
Microvessel density. Values express a qualitative quantification of anatomopathological analysis.

We observed a significant increase in microvessel density in the samples obtained 24h after the supraspinatus muscle injury. (Figure 1)

**Figure 2:**
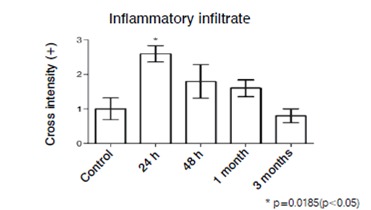
Inflammatory infiltrate. Values express a qualitative quantification of anatomopathological analysis.

Likewise, in the supraspinatus muscle samples collected 24h after the injury there was an increased inflammatory infiltrate (p=0.0185 Kruskal-Wallis-test; p<0.05, Dunn's test). However, in subsequent periods, 48​h and one month after the injury, the inflammatory cell infiltrate, although apparently higher when compared to the control group, did not show statistically significant increase. Interestingly, three months after the injury, the cellular inflammatory infiltrate was very similar to the control group. (Figure 2)

The IHC analysis showed that the proteoglycans chondroitin sulfate and high molecular weight keratan sulphate (aggrecan) and the proteoglycan heparan sulfate (syndecan-1), have not changed during the muscle injury process when compared to the group control.

**Figure 3:**
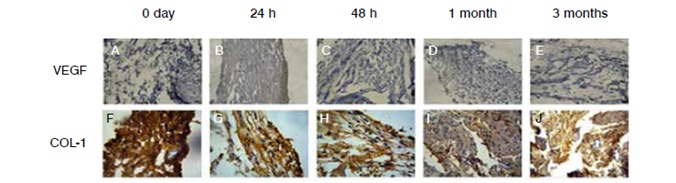
Immunohistochemical reactions for analysis of VEGF and Col-1 expression (A, B, C, D, and E); tissues marked with primary antibody anti-VEGF (F, G, H, I, and J); tissues marked with primary antibody anti-collagen 1.

In our study there were no changes regarding the proteoglycan syndecan-1 24h after the injury, when there was a statistically significant increase in VEGF in comparison to the control group. (Table 1, Figures 3 and ^4^)

**Table 1:** I mmunohistochemical analysis of extracellular matrix components.

Control	24 h	48 h	1 month	3 months
Aggrecan	150.1 ± 6.7	142.4 ± 9.9	164.5 ± 11.1	166.3 ± 16.9	152.1 ± 7.1
*P*	-	0.5556	0.4127	0.7302	1.0000
SYN-1	167.1 ± 1.7	157.9 ± 13.1	166.3 ± 9.2	171.6 ± 5.3	166.7 ± 13.5
*P*	-	0.5714	0.5714	0.5556	1.0000
VEGF	119.8 ± 7.8	143.1 ± 2.7	170.6 ± 33.1	135.6 ± 5.5	145.3 ± 10.7
*P*	-	0.0286	0.4857	0.2000	0.1400
HPSE	140.6 ± 8.3	131.9 ± 7.9	128.7 ± 9.1	153.2 ± 19.4	122.9 ± 3.5
*P*	-	0.5556	0.2857	0.9048	0.2222
Col-1	228.4 ± 5.8	221.5 ± 2.8	218.5 ± 8.2	209.6 ± 7.0	217.3 ± 5.9
*P*	-	0.4127	0.5556	0.0500	0.4127

**Figure 4:**
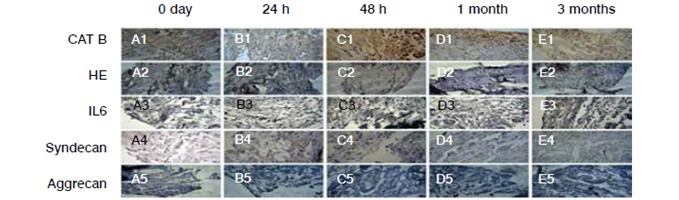
Immunohistochemical reactions for analysis of Cat-B, HE, IL 6, Syndecan, and Aggrecan expression. (A1, B1, C1, D1 and E1), tissues marked with primary antibody anti-Cat- B; (A2, B2, C2, D2, and E2), tissues marked with primary antibody anti-HE; (A3, B3, C3, D3, and E3), tissues marked with primary antibody anti-IL6; (A4, B4, C4, D4, and E4), tissues marked with primary antibody anti-syndecan, and (A5, B5, C5, D5, and E5), tissues marked with primary antibody anti-aggrecan.

The results obtained in this study demonstrated that there was no significant change in the expression of heparanase during the process of the supraspinatus muscle injury in our animal model. When compared to the control group (Table 1 and Figure 4), the results demonstrated that collagen 1 (Col-1) was increased in the samples obtained one month after muscle injury. (Table 1, Figures 1 and ^3^).

The results in Table 2 show that there was no change in expression of Interleukin-6 (IL-6) and cathepsin B (Cat-B) at different times after muscle injury, compared to the control group. (Table 2 and Figure 4)

**Table 2:** Assessment of expression of inflammation markers.

Control	24 h	48 h	1 month	3 months
IL-6	189.5 ± 6.3	200.4 ± 11.1	189.2 ± 11.1	178.1 ± 6.3	177.7 ± 9.8
*P*	-	0.1771	0.9048	0.1905	0.4206
Cat B	205.1 ± 3.5	189.8 ± 19.5	173.2 ± 14.2	193.1 ± 7.4	200.2 ± 11.5
*P*	-	0.5714	0.1905	0.2857	0.5476

## DISCUSSION

Keyes^8^ observed in his study with 192 shoulders of elderly corpses the following changes as the most common: partial tear of the supraspinatus, intratendinous calcification of the supraspinatus, irregularities and hypertrophic changes of the greater tuberosity, adhesion and erosion in the subacromial bursa, bone fragility of the greater tubercle and erosion of the articular surface of the shoulder.

Rathbun and Macnab^9^ observed in a cadaveric stud of injured rotator cuff tendons the formation of a reaction consistent to foreign body reaction and the emergence of a degenerate and avascular tissue in the edge region.

Sher et al.,^10^ in a study using MRI which assessed shoulders of asymptomatic patients, found that 54% of individuals over 60 years old had some rotator cuff injury.

Yamaguchi et al.^11^ conducted a study that evaluated shoulders of 45 patients with asymptomatic rotator cuff injuries over a period of 2.8 years. In 23 shoulders, the injuries progressed to symptomatic injury, and ultrasound evaluation did not show healing in either case, but progression in the severity of injuries in 34% of patients.

Tillander et al.^12^ studied patients with Impingement Syndrome who showed increased level of tissue necrosis and increased concentration of fibrin and fibronectin proportional to the degree of tendon degeneration when subjected to supraspinatus tendon biopsy.

Thomopoulos et al.^13^ in a study that evaluated the extracellular matrix of rats undergoing rotator cuff injury - later repaired - observed an increase in type 1 collagen levels in the tendon and its healing area next to the bone.

Tuoheti et al.^14^ observed up to three times the apoptosis level in the supraspinatus tendon in five patients with stage 2 Impingement Syndrome, compared to the control group (10 cadavers with no history of shoulder pathologies).

Kobayashi et al.^15^ conducted a study in which the shoulders of rabbits were submitted to supraspinatus tendon injury. They found increased levels of IGF and bFGF growth factors in the first week after injury, compared to the control group.

Shen et al.^16^ evaluated 27 patients undergoing arthroscopic acromioplasty and repair of the rotator cuff by mini-open surgery and observed a correlation between the degree of atrophy of the supraspinatus muscle and the functional outcome of the repairs assessed by Constant functional score.

Kim et al.,^17^ in a study with mice, found a relationship between the degree of fatty infiltration, the suprascapular nerve damage and the size of the rotator cuff injury.

Lee et al.^18^ performed MRI exams in 182 patients with rotator cuff injury and observed a relationship between fatty fraction in the rotator cuff injury and the severity of the injury.

Image studies allowed diagnosis and evaluation of the macroscopic evolution of these injuries, regarding increased dimensions, muscular atrophy and fatty infiltration.^10^ ^-^ ^11^ Assessing cadavers with rotator cuff injury showed the formation of avascular and degenerate fibrotic tissue at the edge of injured tendons.^3^ However, imaging studies demonstrated the high evolution capacity of rotator cuff injuries, which did not have spontaneous healing potential.^11^

Studies in humans have shown that tendons from patients with Impingement Syndrome showed increased level of cellular apoptosis and also increased fibrin and fibronectin concentrations, proportional to the degree of tissue degeneration of the tendons. However, studies in rabbits have shown increased growth factors in the extracellular matrix immediately after rotator cuff injury.^5^ In rats, it has been shown increased concentration of collagen-1 in fibrotic scar between the bone and tendon.^1^

We presented here histological and immunohistochemical analysis of the evolution of the supraspinatus muscle injury in Wistar rats, in which we find increased inflammatory infiltrate and microvessel density 24h after the injury. These results suggest that the inflammatory process resulting from the supraspinatus muscle injury is intense up to 24h and some inflammatory response can be observed up to a period of 30 days. However, after three months of muscle injury, the inflammatory process is not shown in the samples. This suggests that, in the rat animal model, intense tissue regeneration occurs 24h after the injury and extends less intensively up to one month.

The immunohistochemistry analysis allowed us to evaluate various components of the extracellular matrix such as proteoglycans aggrecan and syndecan-1, VEGF, HPA enzyme and Col-1, comparing samples of injured supraspinatus muscle with control samples of uninjured muscle. (Table 1) It has been shown that tendon aggrecan increased after injury of the supraspinatus muscle, further indicating that this increase occurs in the tendon insertion site and precedes the mechanical structural changes and muscle changes.^19^

We noticed that the aggrecan is not changed in the supraspinatus muscle in the post-injury period. Therefore, the results obtained in this study differ from those described in the literature, possibly because different tissue samples and also different periods were evaluated. Also in this regard, it is important to emphasize that the constitution of the supraspinatus tendon varies across the tissue.^20^

It has been extensively described in the literature modulation of angiogenesis by syndecan-1 proteoglycan. However, our study showed no increase of syndecan-1 in periods of higher angiogenesis.^18^

VEGF showed increase 24h after injury, which can be explained by tissue hypoxia that occurs after injury. This stimulates the vascular neoformation process in the region mediated by various cytokines, including VEGF, which performs an important role in stimulating early vascular neoformation, which starts after a few hours after tissue injury.^19^

The HPA enzyme is an endo-beta-glucuronidase that specifically degrades heparan sulfate chains and heparin. Such enzyme is increased in some inflammatory processes and carcinogenesis. However, we observed in our study no heparanase change in the evolution of tendon injury.^20^

Col-1 is the most abundant collagen in tendons. Studies have shown that in the early phases of the tendon healing process - the inflammatory and proliferative phases - occurs a predominance of type 3 collagen in the region where the scar is formed. However, in the remodeling phase, around four to eight weeks after injury, Col-1 is increased, as found in our study, and there is also a statistically significant difference in four weeks compared to the control group. This increase in Col-1 concentration allows the fibrotic scar to partially restore biomechanical properties of the original tissue, since Col-1 is more resistant to tensile forces than type 3 collagen.^5^

Literature data show that IL-6, a pro-inflammatory cytokine, is increased in posterior tibial tendon and Achilles tendons from patients who had severe pain as compared to tendons of patients who showed no signs of disease, even when the signs of inflammation were not evident.^20^ In our study we observed increased inflammatory infiltrate 24h after injury. However, IL-6 concentration did not show any changes, probably because the evaluation period of the animal model did not favor IL-6 study. We must also consider that such studies evaluating IL-6 were performed in different tissues of different origin, which may explain the differences regarding the results of IL-6 expression.

Cathepsins belong to the family of cysteine ​​proteases; most of them act at low pH and their activity can be observed in lysosomes. However, cathepsin K is an exception because after its secretion, this protease is enzymatically active in the extracellular matrix, and such role is essential in the process of bone resorption.^19^

It has been found a significant increase in the enzymatic activity of cathepsin L and cathepsin K four and eight weeks after rotator muscle injury in the tendon of the supraspinatus muscle.^19^

In the present study we decided to investigate Cat-B because it is protease that actively participates in the inflammatory process.^20^ We found no change in this protease concentration in any period of our study.

## CONCLUSIONS

After injury to the supraspinatus muscle there are changes in the components of the extracellular matrix, which account for remodeling and regeneration processes. Such processes are more intense 24h after injury and characterized by severe inflammation and increased microvessels. Among the components of the extracellular matrix evaluated, VEGF and Col-1 increased significantly 24h and one month, respectively, after the supraspinatus muscle injury.

## References

[1] Fukuda H (2000). Partial-thickness rotator cuff tears a modern view on Codman&apos;s lassic. J Shoulder Elbow Surg.

[2] Gartsman GM, Milne JC (1995). Articular surface partial-thickness rotator cuff tears J Shoulder Elbow. Surg.

[3] Ikemoto RY, Murachovsky J, Nascimento LGP, Bueno RS, Almeida LH, Strose E (2007). Avaliação da microcirculação das bordas do tendão do supra-espinal nas lesões do manguito rotador. Rev Bras Ortop.

[4] Nakagaki K, Ozaki J, Tomita Y, Tamai S (1996). Fatty degeneration in the upraspinatus muscle after rotator cuff tear. J Shoulder Elbow Surg.

[5] Steinbacher P, Tauber M, Kogler S, Stoiber W, Resch H, Sänger AM (2010). Effects of rotator cuff ruptures on the cellular and intracellular composition of the human supraspinatus muscle. Tissue Cell.

[6] Soslowsky LJ, Carpenter JE, DeBano CM, Banerji I, Moalli MR (1996). Development and se of an animal model for investigations on rotator cuff disease. J Shoulder Elbow Surg.

[7] Matos LL, Stabenow E, Tavares MR, Ferraz AR, Capelozzi VL, Pinhal MA (2006). Immunohistochemistry quantification by a digital computer-assisted method compared to semiquantitative analysis. Clinics (Sao Paulo).

[8] Keyes EL (1935). Anatomical observation on senile changes in the shoulder. J Bone Joint Surg Am.

[9] Rathbun JB, Macnab I (1970). The microvascular pattern of the rotator cuff. J Bone Joint Surg Br.

[10] Sher JS, Uribe JW, Posada A, Murphy BJ, Zlatkin MB (1995). Abnormal findings on agnetic resonance images of asymptomatic shoulders. J Bone Joint Surg Am.

[11] Yamaguchi K, Tetro AM, Blam O, Evanoff BA, Teefey SA, Middleton WD (2001). Natural istory of asymptomatic rotator cuff tears a longitudinal analysis of asymptomatic tears detected sonographically. J Shoulder Elbow Surg.

[12] Tillander B, Franzén L, Norlin R (2002). Fibronectin, MMP-1 and histologic changes in rotator cuff disease. J Orthop Res.

[13] Thomopoulos S, Hattersley G, Rosen V, Mertens M, Galatz L, Williams GR (2002). The localized expression of extracellular matrix components in ealing tendon insertion sites an in situ hybridization study. J Orthop Res.

[14] Tuoheti Y, Itoi E, Pradhan RL, Wakabayashi I, Takahashi S, Minagawa H (2005). Apoptosis in the supraspinatus tendon with stage II subacromial impingement. J Shoulder Elbow Surg.

[15] Kobayashi M, Itoi E, Minagawa H, Miyakoshi N, Takahashi S, Tuoheti Y (2006). Expression of growth factors in the early phase of supraspinatus tendon healing in rabbits. J Shoulder Elbow Surg.

[16] Shen PH, Lien SB, Shen HC, Lee CH, Wu SS, Lin LC (2008). Long-term functional outcomes after repair of rotator cuff tears correlated with atrophy of the supraspinatus muscles on magnetic resonance images. J Shoulder Elbow Surg.

[17] Kim HM, Galatz LM, Lim C, Havlioglu N, Thomopoulos S (2012). The effect of tear size and nerve injury on rotator cuff muscle fatty degeneration in a rodent animal model. J Shoulder Elbow Surg.

[18] Lee S, Lucas RM, Lansdown DA, Nardo L, Lai A, Link TM (2015). Magnetic resonance rotator cuff fat fraction and its relationship with tendon tear severity and subject characteristics. J Shoulder Elbow Surg.

[19] Legerlotz K, Jones ER, Screen HR, Riley GP (2012). Increased expression of IL-6 family members in tendon pathology. Rheumatology (Oxford).

[20] Theodoro TR, de Matos LL, Sant Anna AV, Fonseca FL, Semedo P, Martins LC (2007). Heparanase expression in circulating lymphocytes of breast cancer patients depends on the presence of the primary tumor and/or systemic metastasis. Neoplasia.

